# Indents

**Published:** 1922-10

**Authors:** 


### INDENTS

**☞Brief Articles of Technical or Scientific Value will receive notice and comment. Contributions invited.**

---

Inlay Wax. When Dr. Taggart first gave us a special wax for inlay technic he made it purposely very hard. A few men who secured generous lots of that wax are still using and hoarding it carefully, and keeping it for special uses. Dr. Taggart was asked recently why he did not continue to make it of the same consistency, he said, "When I was manufacturing wax by my original formula, another manufacturer put out waxes of various grades, but they all advertised soft and pliable waxes, and sold hundreds of boxes to one of mine, because his waxes were easier to manipulate than mine. It is true the hard wax was better even though more difficult to manipulate. The dentists were not looking for the best results in inlay technic, but rather they sought the easier way of making inlays. To meet this demand and give the dentists what they supposed they wanted, rather than what they ought to have had, I was forced to change my formula and make my waxes softer. I never made it as soft as other manufacturers, but yet considerably softer than it should have been. Nowadays as men have grown more skilful in the inlay technic there is a continued demand for a harder wax; waxes that will make sharper impressions without crawling when properly manipulated; in fact a wax that softens at a higher temperature and holds its shape in the warmed mouth, and afterward while awaiting the convenient hour for investment. Of course I shall make the old and harder wax if there is ever a sufficient demand for it. As this is a fair proposition, why not take advantage of this offer." If a sufficient number of dentists would care for enough wax of this kind, to justify his making it, Dr. Taggart will be glad to make it up as a special order.—Dr. Ottolengui, in *Around the Table.*

NOMENCLATURE. List of words recommended by the Committee on Dental Nomenclature, American Dental Association.

alveolectomy (L. alveolus + Gr. ektome, excision). Excision of a portion of the alveolar process.

alveolotomy (L. alveolus + Gr. tome, from temnein, to cut). Incision into the alveolus of a tooth, as for locating the end of a root of a tooth.

anesthesia. Preferable to anaesthesia.

apicoectomy (L. apex, gen. apicis, the end [of a tooth root] + Gr. ektome, excision). The operation of excising the end of the root of a tooth. To be used in preference to apectomy; apiectomy; apicectomy.

artificial denture. Preferable to plate.

cuspid. To be used in preference to canine.

cementum. To be used in preference to cement.

conduction. To be used in preference to conductive, as in conduction anesthesia.

deciduous (adj.). To be used as designating the teeth of the first dentition, in preference to the terms ''temporary,'' ''milk'' or ''baby.''

dentural (adj.) (L. dens, dentis, tooth). Relating to the denture.

first molar. To be used in preference to ''six-year molar,'' ''sixth-year molar.''

mandible (L. mandibula from mandere, to chew). The lower jaw.

maxilla, pl. maxillae (L. maxilla, jaw). The upper jaw.

morsal and occlusal (adj.). To be used synonymously as relating to the masticating surfaces of the bicuspid and molar teeth.

centric occlusion. To be used to express the relation of the inclined planes of the teeth when the jaws are closed in the position of rest.

eccentric occlusion. To be used to express the relation of the inclined planes of the teeth in the excursive movements of the mandible.

mesial and distal. These terms as used today have been objected to as not being in conformity with anatomical nomenclature, where they are used to indicate relation to the median line of the body. They have, however, become so fixed in dental nomenclature that we do not suggest any change.

pathodontia (Gr. pathos, disease + odous, tooth). That branch of dentistry which has for its purpose the study and treatment of diseases of the teeth.

pathology (Gr. pathos, disease + logos, treatise). That branch of medical science which treats of morbid conditions, their causes, symptoms, etc. This term is being loosely used to indicate a disease or pathologic condition, which is confusing, unnecessary and undesirable.

pediadontia (Gr. pais, paidos, child + odous, tooth). That branch of dentistry which has for its purpose the study and treatment of children's teeth and mouth conditions.

periodontia (Gr. peri, around + odous, tooth). That branch of dentistry which has for its purpose the study and treatment of diseases occurring around the teeth and their roots.

periodontal (Gr. peri, around + odous, tooth). Relating to the alveolo-dental ligament. To be used in preference to peridental.

periodontoclasia (Gr. peri, around + odous, tooth + klassis, breaking [down]). The destructive degeneration of the tissues about the root of a tooth. Substituted for pyorrhea alveolaris; Rigg's disease; interstitial gingivitis.

periclasia (Gr. peri, around + klassis, breaking [down]). Used as a shortening for convenience of periodontoclasia. Should be used with a qualifying word, as in itself it does not mean anything in particular.

pontic (L. pons, pontis, a bridge). (adj. and noun.) A substitute for a natural tooth. Used in preference to dummy.

bicuspid. To be used in preference to premolar.

prosthesis (n.) (Gr. pros, to + tithenai, to place). Preferable to prothesis. (Because of the more definite application of the Greek preposition pros, as compared to pro, in this form.)

prosthetics (n.). Preferable to prothetics. (For same reason as in prosthesis.)

pulpless tooth. To be used in preference to "dead tooth," "devital tooth," "devitalized tooth." In cases where there is a "vital" pulp in a tooth or a "non-vital" pulp, it should be so designated; e. g., a tooth with a vital pulp, or a tooth with a non-vital pulp.

radiology (n.) (L. radius, ray + Gr. logos, treatise). The science of radiant energy. To be used as the generic term to indicate radiant energy from whatever source.

radiogram (n.) (L. radius, ray + Gr. gramma, a writing). The product or tangible result, as the film or the print thereof, of the radiographic process, actuated by radiant energy of whatever source.

radiograph (verb) (L. radius, ray + graphein, to write). The act or process of making a radiogram.

radiography. The art of making radiograms.

radiopaque (L. radius, ray + opacus, shady). Term applied to a substance that is impermeable to the various forms of radiant energy.

radiolucent (L. radius, ray + lucere, to shine). Term applied to substances that allow the passage of radiant energy light, but offer some resistance.

radioparent (L. radius, ray + parere, to appear). Term applied to substances that freely transmit the light of radiant energy.

Roentgen ray. To be used in preference to X-ray, and only where the specific ray is indicated.

roentgenology. The study and use of the Roentgen ray in its application to medicine and dentistry.

roentgenography. The art of making roentgenograms.

roentgenogram. The shadow picture produced by the Roentgen ray on a sensitized film, or the print from the film.

roentgenograph (v.). The act of making a roentgenogram.

second molar. To be used in preference to "twelve-year molar," or "twelfth-year molar."

third molar. To be used in preference to "wisdom tooth."

Vincent's infection. To be used to express the ulceromembranous stomatitis caused by Vincent's spirillum and fusiform bacillus; in preference to Vincent's angina; the latter being more applicable to the throat infection.

x-ray (n.). This word is used indiscriminately as a noun and verb. It should not be used as a verb. The word Roentgen ray is preferable. It should also be used with small x rather than with the capital X, if used at all.

penetology; odontalysis. These two words have been suggested, the first to mean the science of radiant energy, and the latter, examination of the teeth. We see no justification for these two words either etymologically or otherwise.

---

What is the Value of a Deciduous Tooth? The answer to the above question may be found in the following excerpts, taken from a recent article by Dr. E. E. Graham, Lake Forest, Ill., and appearing in the "Dental Summary," June, 1921:

1. Premature loss of the deciduous teeth is the cause of from 85 to 95 per cent. of all irregularities of the human arch.

2. Premature extraction of a deciduous tooth is followed by the regeneration of bony tissue, which forms a dense layer, resisting the eruption of the permanent tooth.

3. Each deciduous tooth should be retained until time for the eruption of its successor, but it is most important that the deciduous second molars and cuspids be retained to guide the first permanent molars and cuspids into their positions. A deciduous cuspid should never be removed to make room for a crowded lateral incisor.—Dr. J. E. Gurley, in *Pacific Gazette*.

---

The Gangrenous Pulp in a Deciduous Tooth. This is one of the usually easy cases handled. Open into the pulp chamber. Wash out with warm water applied with a syringe. The tooth may be left open for the first twenty-four hours, if desirable, though it is not advisable, for with Buckley's formo-cresol the gas formation will be reduced, and then, too, the first step in sterilization will be accomplished. This treatment should be changed at the end of twenty-four hours, when, if necessary, it may be repeated for another similar period. Usually at the end of forty-eight hours, at the most, the canals may be cleansed by means of canal cleansers and washed with hydrogen dioxide. After drying, I prefer to carry a weaker disinfectant for a few days, as a matter of security, or it may not be convenient to continue the cleansing process at the time of discontinuance of the formo-cresol remedy, in which case also I use a weaker drug. I have found one recommended by Grove to be very satisfactory, the formula of which is as follows:

Chloral hydrate.
Thymol, equal parts.
Alcohol, sufficient to keep it liquid.

This dressing may be later removed, the canals dried and filled with a paste, as follows:

| | |
|---|---|
| Zinc oxide | 15.00 |
| Xeroform | 5.00 |
| Phenol | gtt. XXX |
| Glycerine | q. s. |

—J. E. Gurley, in *Pacific Gazette.*

---

STERILIZING PYORRHEA POCKETS. In regard to the necessity of removing necrotic membrane or tissue, it is a positive necessity. I have attained the best results by the use of the electric cautery; its use so far as the patient is concerned, it is more agreeable to use the cautry than the knife. In chronic cases it is very often expedient to use the cautery at the first sitting.

In reference to the control of the patient, the necessity of which we all realize and the importance of which was very forcibly impressed on me about twenty-five years ago, I wish to relate the following incident: While visiting the late D. D. Smith, of Philadelphia, a patient came in whom he had repeatedly reproved for not following his instructions. Feeling that it was useless to persist, he summarily discharged her, notwithstanding the fact that her case was not completed. Although the procedure, at that time, was considered a little radical, I have since concluded that Dr. Smith was justified in discharging the patient. Without your patient's hearty co-operation you can accomplish very little.

Ionization is another method of stimulating cell life, but before deciding to treat a patient by ionization it is advisable to pause and consider what the component factors of the patient's condition are. In a septic area, for example, we should ask, are the tissues infected by a bacterium? Are they irritated by a well of putrefying discharge? Is this well mechanically accessible in its whole extent? If the tissues are infected, treatment by a vaccine or serum is rational. If there is a putrefying well it is also rational to wash this out, fill the cavity with zinc solution and ionize the surface layers of the wound—K. S. Perry, in *Journal A. D. A.*

---

A NEW IDEA IN COMMUNITY DENTAL SERVICE. There has recently been inaugurated in the City of Chicago a plan whereby the teeth of children may be adequately cared for without taxing private philanthropy or relying on civic appropriations. The profession is familiar with the splendid Forsyth Dental Infirmary of Boston, and the Eastman Dental Dispensary of Rochester. Dentists in the vicinity of Chicago also know of the generous assistance given to school dentistry in this city by Mr. Julius Rosenwald. In other sections of the country civic bodies have been induced to support this work, and everywhere there has been an awakening as to the urgent need of constructive measures for the amelioration of conditions existing in the mouths of the children of the nation.

But today Chicago has a plan whereby the need is to be met in a very unique way. The funds, instead of coming from private individuals or from civic bodies, are to come from the community, and the money is to be raised in such a way that the community will never feel the burden, and as a matter of fact it will be entertained and benefited while it is furnishing the funds.

In 1921, Mayor William Hale Thompson, with the aid of such men as Dr. John Dill Robertson, then commissioner of health, organized what was called the

Pageant of Progress—an exposition illustrative of the development of the various arts, sciences, and industries in Chicago, as an instructive and educational movement whereby the masses might be made familiar with the growth and present status of the different industrial activities in their midst. This pageant was held during the summer of 1921, and turned out such a success not only in an educative way but also in its financial returns that a fund was created of approximately $150,000 after all expenses were paid.

It is now the purpose to make this pageant an annual event, and to utilize the money so raised for the building and maintenance of a dental infirmary for the care of the needy children of Chicago. It will thus be seen that the philanthropic and utilitarian features of this plan fit in so perfectly that it promises to be the solution of what has always been a difficult problem.

Dr. Robertson has been made manager of the pageant, having resigned as commissioner of health for this purpose, and under his active and fertile brain there can be no question of the continued success of the enterprise.

At the Los Angeles meeting of the National Dental Association, Mayor Thompson sent a communication, on invitation of the president and secretary of the association, outlining the plans for this great philanthrophy, and enthusiastically assured the association that ground would probably be broken for the building not later than October of the present year.

This seems one of the most concrete and far-reaching plans ever devised for meeting the urgent need presented by the large percentage of children who are today suffering from dental neglect, and it is a movement which, in the very nature of its character, must ultimately draw to it the hearty support of every dentist in the land and of every worthy citizen of Chicago.—*Journal A. D. A.*, August, 1922.

Root Canal Fillings. Probably root canal operations were not made very commonly fifty or sixty years ago, but I am convinced that they became common very soon after that. In the "Cosmos," Vol. III, 1861-62, page 556, there is reported a case in which Dr. Hudson of Philadelphia had filled the root canals of the upper centrals with gold about 1830, and they were in perfect condition at the time of the report. In the "News Letter," Vol. X, 1856-7, page 4, Dr. J. S. Clark of New Orleans is quoted as saying that he had "practiced fang filling with gold for the past nine or ten years." He said the profession knew how far Koecker had carried his operations in this direction, and "Maynard, Harwood, Badger and Hudson practiced it years before any of us." In the same volume, page 234, Drs. Spaulding, Taft, Watt and others report success with gold as a root-filling. Possibly these reports imply a more general practice of filling roots in the first half of the last century than I suppose. It is my belief that such operations were made only by a very few of the best and most progressive operators and probably they were almost entirely limited to the upper front teeth.

Dr. A. D. Black cites several articles mentioned, together with those of about 1860, which indicate that the treatment of pulps was not seriously undertaken by the profession previous to 1865, and that after 1870 the attempts to save teeth with exposed living pulps, or with dead pulps, became quite common practice, the method generally employed being the removal of the contents of the canal and placing of from one to many treatments of creosote on cotton, most operators leaving such a treatment as a root filling. Quite a number of the better operators used gold foil for root filling. The first mention I found of a gutta-percha root filling was in the "Cosmos," Vol. XIV, 1872, page 371, in an article by Dr. Oscar Doyle, of Louisville, Ky.

I am surprised that no one wrote about filling pulp canals with gutta-percha before 1872. A little history personally known to me will explain why. When I entered the office of Dr. E. L. Clarke, in Dubuque, Iowa, as a student, in May, 1865, he was filling the roots of teeth with base-plate gutta-percha, which, as I remember, was the identical material that we know by that name now. His method was to heat it as hot and fluid as possible without burning it, and churn it into the pulp canals with a hot instrument. I happen to remember one case for which I held a little broken piece of crockery containing the material, with a pair of pliers over the alcohol lamp till it was just ready to burn, and standing beside the chair while he filled the roots of a lower molar. The material in that state is very plastic and very sticky. I have no knowledge of how long he had been doing that or of whom he learned the method. I have reason to suppose that his operations were very successful. I believe it was this use of a hot instrument by my preceptor that saved me from ever making the cold operations that became so common soon after, filling a canal with chloro-percha and thrusting into it a cone of hard gutta-percha. It is my belief that this method has been responsible for the large number of abscesses following root canal operations and the bad name the profession got for this part of our work. A great number of such operations have been successful, but most of you have taken enough of these hard points out of stinking canals to justify my opinion.

I used chloro-percha myself for a time but always followed it by additional gutta-percha made plastic with heat and worked in with a hot instrument. It was not very long before the ''pure volatile eucalypti extract'' of Sander and Son, Australia, was substituted for the chloroform, and with that I used, and have ever since, the ''Hill's Stopping'' preparation of gutta-percha. For a good while I have used Dr. Buckley's eucalyptol compound. This method was not original with me and I do not know who first advised it. Dr. A. E. Matteson was one of the early users of it. Perhaps it was from him I learned it. This method has served me so well that I have continued it to the present time and all of the root fillings the history of which I will presently give you were made in this way. I will not advise it in competition with the method described and skilfully used by Dr. Gethro, Dr. Coolidge and others, or the Callahan method of infiltrating the dentin with a solution of rosin, or, perhaps, the precipitation of silver, if the formalin used can surely be kept away from the apical space.

Many different materials have been proposed, and to some extent used for filling pulp canals. In the old days some men very successfully used tin foil instead of fold foil. Gold, silver, copper and lead wires have been used by some operators. Dr. J. Foster Flagg used and persistently advocated cotton and creosote and affirmed that he smelled the creosote on removing some of his fillings years afterward. Other dentists removed a good many of them for the relief of trouble and smelled something quite different. Dr. J. N. Crouse filled pulp canals successfully with oxychloride of zinc and he believed that material would be effectively antiseptic for a long time. Some have tried to use the ordinary oxyphosphate cement, with how much success or satisfaction I do not know.

About fifty years ago Dr. Kennicott and some others advised what they called "knocking out" the living pulps of upper front teeth with a tapering hickory stick, struck a pretty sharp blow with a mallet. The pulp was expected to come away with the stick, and the same stick, or a similar one fitted more carefully, was sometimes used to fill the canal. It was claimed that this procedure was not very painful, but it is my belief that a large proportion of the men who tried it once did not do it any more.

There is one thing I want to say in regard to these fillings that do not reach the ends of the canals. It is my belief that we should in every case, wherever we can possibly do so, get our filling to the end of the pulp canal. In every one of those cases in which I did not get there, you may depend I spent an hour or more, maybe two or three, in trying to get there. But the point I want to make particularly is that we may ignore absolutely the foramen through the cementum if we can fill the pulp canal to the end of the dentin. While you can not fill a minute foramen, which is as fine as a hair in some cases, in my judgment a tooth is three or four or ten times as safe if you fill the root to the end of the pulp canal, leaving the fine foramen through the cementum of the root without meddling with it, as it would be if you drilled through the foramen and carried the filling to the end of the root and put a cap over the end of it, as Dr. Rhein advocates.

In regard to partially filled canals, Dr. Hatton said that he had found a small amount of living tissue in a few roots that had carried root fillings part way down and fillings in the crowns for a long time. Until he actually found this, none of us believed it would ever happen. I do not think it happens very often, but it may account for the success of a few of these cases in which we can not deny the impossibility of getting our fillings to the end, provided we have not poisoned the tissue with arsenic beforehand.

We honor Dr. Flagg for his sincerity, his earnestness and great work in the profession, although some of us think his work was injurious. I have no doubt that a great many cotton fillings he made were successful, but I have seen a considerable number of other men who have taken out some of Dr. Flagg's cotton fillings and did not smell creosote. They were taken out for the relief of trouble in the teeth. I have done the same with a lot of teeth filled with thin chloropercha and a round cone of hard gutta-percha thrust into it. I do not think a great many of them were as good as the solidly packed cotton fillings. I have had a good many cases come to me that were giving trouble and have taken out the fillings and found the roots very foul indeed.—Edmund Noyes, in *Journal A. D. A.*

---

SCARE-ADVERTISING. It was the late Phineas T. Barnum, we believe, who was the author of the statement that "the public likes to be fooled." While in many ways the world has made great progress since Barnum's characterization of the public taste was uttered, there is ample evidence pointing to the conclusion that the public fondness for certain kinds of deception is as keen as ever and that the business of meeting the public demand in that regard still goes merrily on.

Barnum's "fooling" of the public was in the nature of providing wholesome entertainment and in that respect was not only harmless but to a large decree salutary. There is, however, a phase of this "fooling" that is vicious and harmful, without benefit to anyone excepting in a monetary way to those who exploit the ignorant and impressionable element of the public for financial gain by means of misleading advertising of a quasi-medical and scientific character. We refer particularly to the methods and activities of the ubiquitous and omnipresent quack nostrum vender.

For many years in the past these gentry confined their operations almost wholly to the medical field, they flooded the country with preposterous claims for worthless preparations, guaranteed to cure any or all of the ills that flesh is heir to, at prices which, compared with the actual value of their wares, were extortionate. All of which has become matter of common knowledge, so that today only the ignorant and superstitious are impressed by the claims of the medical quack. With the advance of medical knowledge, however, a new phase of "fooling" the public has arisen. The discovery that the infected mouth is the source of a variety of systemic or metastatic disorders has been seized upon by the genus "quack" as a new and fruitful field for exploitation, so that with a certain background of ascertained scientific facts a new crop of the breed of quacks has arisen and invaded the dental field. This new variety might be properly designated as the terror-monger, the analogue of his medical prototype who thrives upon the fruits of his scare-advertising of nostrums for the cure of impotence, cancer, etc.

Dental research has developed certain well-known and accepted facts, e. g.,

A. That the mouth is a harbor and incubating ground for bacteria, many varieties of which are pathogenic.

B. That certain bacteria cause dental caries.

C. That the mucinous plaque is the agent responsible for the fixation and localization of caries-producing bacteria and that the plaque may be dissolved by alkalies or coagulated by acids and astringents.

D. That pyorrhea is a disease of germ origin.

E. That germs developing in or about the teeth may be transferred to other parts of the body and there establish disease foci.

F. That the constant swallowing of toxins and bacterial waste products is productive of ill-health.

G. That the reaction of the oral fluids may normally vary from neutral to alkaline or acid within the limits of health and that such variations are therefore not pathologic in themselves nor are they productive of pathologic reactions.

Each one of these facts has been seized upon by promoters of nostrums in the shape of dentifrices which are exploited as cure-alls for dental disease and preventives of all the bodily ills therefrom arising.

As an offset to these claims in general we submit that dental research has not up to this time devised any method whereby the mouth cavity can be safely or successfully sterilized. Sterilization as applied to the oral cavity is purely a relative term meaning that by a variety of means the bacterial content may be appreciably diminished for an appreciable period of time. Research has further shown that no active germicide sufficiently powerful to destroy mouth bacteria has been discovered or that can be used continuously for mouth sterilization without damage to the oral tissues. In view of the solvent action of alkaline and saline solutions on the bacterial plaque, as well as the coagulating action of acids and astringents thereon, it is still an open question which is the more efficacious method, and the extravagant claims of superiority put forth by venders of dentifrices of either class are without scientific justification. As the reaction of the oral fluids may normally vary from time to time within the limits of physiological health the reaction by litmus test is valueless as an indication of a dangerous pathological state or as being an indication of an existing degree of acidity that is a menace to the integrity of the dental structures.

The terror-mongers by seizing upon a few accepted scientific facts and distorting them with figments of their imagination have created a situation in the public mind that may well give us pause and not only food for serious consideration, but grounds for decisive action as a self-respecting profession.

Rudyard Kipling reports an interview with the late Mark Twain in which the factors of a successful literary training were under discussion, concerning which the latter said, that in his opinion the surest way to success as a fiction writer was to store one's mind with facts and you could then distort them at your leisure—which is all very well for avowed purposes of amusement, but the public health and public confidence are not matters to be legitimately trifled with and certainly not for commercial gain. It is but fair to grant that a proportion of the advertising claims for these cure-all dentifrices are based upon insufficient knowledge and with an honest intent to popularize an intrinsically good thing, but there is also manifest in a goodly proportion of dentifrice advertising the tactics of the quack and terror-monger, who prey upon the credulity and fears of the ignorant and superstitious. It is they whose dishonest practices and impossible claims should be suppressed in the interests of public health and public morals, for they constitute the modern exemplification of St. Mark's prophecy, that "false prophets shall rise and shall shew signs and wonders to seduce, if it were possible, even the elect."

The medical profession has for years waged effective warfare against these excrescences upon the fair name and reputation of their professional calling. It would seem that the time is full ripe for a dental crusade in the same direction.—*Dental Cosmos*, October, 1922.

